# The use of nanoparticles for targeted drug delivery in non-small cell lung cancer

**DOI:** 10.3389/fonc.2023.1154318

**Published:** 2023-03-09

**Authors:** Jessica E. Holder, Christopher Ferguson, Elisabete Oliveira, Carlos Lodeiro, Carol M. Trim, Lee J. Byrne, Emilia Bertolo, Cornelia M. Wilson

**Affiliations:** ^1^ Canterbury Christ Church University, School of Psychology and Life Sciences, Life Sciences Industry Liaison Lab, Sandwich, United Kingdom; ^2^ BIOSCOPE Research Group, Laboratório Associado para a Química Verde- Rede de Química e Tecnologia (LAQV- REQUIMTE), Chemistry Department, NOVA School of Science and Technology, Universidade NOVA de Lisboa, Caparica, Portugal; ^3^ PROTEOMASS Scientific Society, Madan Parque, Rua dos Inventores, Caparica, Portugal

**Keywords:** nanoparticles, lung cancer, drug delivery, therapeutics, cancer

## Abstract

Lung cancer is a global health problem affecting millions of people each year. Non-small cell lung cancer (NSCLC) is the most common form of lung cancer with various conventional treatment available in the clinic. Application of these treatments alone often results in high rates of cancer reoccurrence and metastasis. In addition, they can cause damage to healthy tissues, resulting in many adverse effects. Nanotechnology has emerged as a modality for the treatment of cancer. When used in combination with nanoparticles, it is possible to improve the pharmacokinetic and pharmacodynamic profiles of pre-existing drugs used in cancer treatment. Nanoparticles have physiochemical properties such as small size which allowing passage through challenging areas of the body, and large surface area allows for higher doses of drugs to be brought to the tumor site. Nanoparticles can be functionalized which involves modifying the surface chemistry of the particles and allows for the conjugation of ligands (small molecules, antibodies, and peptides). Ligands can be chosen for their ability to target components that are specific to or are upregulated in cancer cells, such as targeting receptors on the tumor surface that are highly expressed in the cancer. This ability to precisely target the tumor can improve the efficacy of drugs and decrease toxic side effects. This review will discuss approaches used for targeting drugs to tumors using nanoparticles, provide examples of how this has been applied in the clinic and highlight future prospects for this technology.

## Introduction

Cancer is a leading cause of death worldwide, accounting for approximately 1 in 6 deaths (1). Compared with other cancer types, lung cancer has one of the poorest survival outcomes with approximately 1.8 million deaths annually (2). Based on the histology of the cancer cells, lung cancer is classified as either non-small cell lung cancer (NSCLC) or small cell lung cancer (SCLC). NSCLC is the most common form and accounts for over 85% of all cases of lung cancer. NSCLC can begin in various types of epithelial cells that line the lungs, whereas SCLC will generally always begin in the bronchi (3). Currently, diagnosis and staging of lung cancer can involve chest radiographs, computed tomography (CT) scans, biopsies, and positron emission tomography (PET) scans (4). Conventional treatment for NSCLC includes surgery, which can involve removing part of the lung or the whole lung, followed by chemotherapy and/or radiotherapy (5). An alternative treatment which is less widely used is immunotherapy; using antibodies to trigger the immune system into targeting the cancer cells. Monoclonal antibodies (MABs) can be designed to bind to target proteins on cancer cells so they can be more easily detected by the immune system. MABs are also used as checkpoint inhibitors which target and block checkpoint proteins on immune cells to enhance the immune response against the tumor (6). Patients with NSCLC are often tested for molecular markers; changes in genetic sequences, gene expression levels and protein structures and functions, associated with disease sub-types and stages (7). These markers can be used to help diagnose and stage diseases and provide prognostic information. In cancer therapy, molecular markers can also be used to predict how patients will respond to certain treatments, allowing treatment plans to be personalized to each patient to increase the efficacy of treatment. NSCLC patients will most commonly be tested for mutations in epidermal growth factor receptors (EGFR), anaplastic lymphoma kinase (ALK) and Kirsten rat sarcoma viral oncogene homolog (KRAS). EGFR is overexpressed in 62% of cases of NSCLC and patients with this mutation have high response rates to treatments with tyrosine kinase inhibitors; therefore, this has become the standard treatment for patients with this mutation (8)

Rearrangement of the ALK gene is detected in around 4% of NSCLC patients and these patients tend to be highly responsive to ALK inhibitors (9). Lastly, KRAS mutations occur in 25 - 35% of NSCLC cases. Currently, there are no clinically approved drugs to target KRAS mutations, however, this marker can still inform a patient’s treatment plan as it is a negative predictor of response to chemotherapy, but evidence suggests that cancer with this mutation is more vulnerable to immunotherapy (10, 11)

The rate of recurrence in NSCLC is between 30% and 55% depending on the stage of the disease – highlighting the need to refine current treatments and develop new methods to treat it (12). Conventional treatments can also damage nearby healthy tissue, resulting in several adverse effects for patients. Using nanotechnology, it is possible to develop new methods to target drugs to tumor sites, increasing the efficacy of the drug and reducing the harmful effects on healthy tissue.

Nanotechnology describes science, engineering and technology that works with particles that are on the nanometer scale called nanoparticles (NPs). NPs can be made up of carbon, metal, metal oxides or organic matter and can be engineered to have different shapes and sizes as well as altered surface chemistries; allowing several types of NPs to be produced with various biological applications (13). The physiochemical properties of NPs (such as their small size) permit passage through challenging areas (such as the blood-brain barrier) while the ability to modify their surface chemistries allows the NP-drug conjugate to be targeted to tumor cells. Several NPs are therefore being developed to deliver therapeutic agents to tumor cells, including in NSCLC.

## Current uses of NPs in the clinic

**Figure 1:**
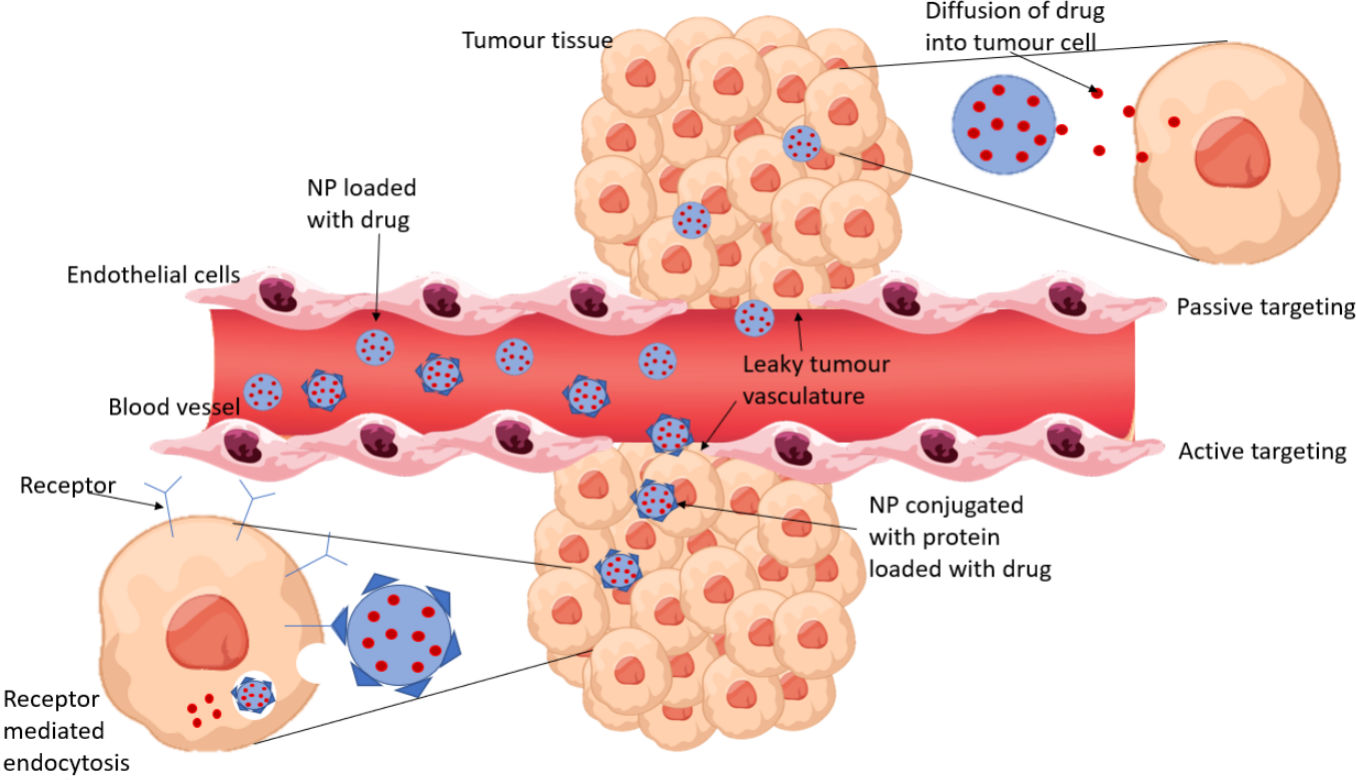
A comparison of passive and active drug targeting. Passive targeting relies on the EPR effect for NPs to accumulate in tumor tissue and release the encapsulated drug to the cells by diffusion. Active targeting involves conjugating proteins to the NPs which bind to receptors that are overexpressed by tumor cells. The NP enters the tumor cell by receptor mediated endocytosis and the drug is released.

Nanoparticles can be passively targeted to tumor cells due to the enhanced permeability and retention effect (EPR). This describes how nanoparticles will preferentially accumulate into tumor tissue (due to the tumor’s leaky vasculature) and will remain in these tissues for prolonged periods as they have impaired lymphatic drainage. The EPR effect means drug-loaded NPs will concentrate high drug doses into tumors compared with the free drug and therefore decrease exposure to healthy tissue (14) Passive and active drug targeting is represented in **Figure 1**.

Currently, two nanoformulations have been approved for clinical use in the treatment of NSCLC – Abraxane and Genexol-PM. Both drugs use the same active substance, paclitaxel (PTX) which prevents microtubule dissociation and so inhibiting mitosis leading to cell death. PTX is highly lipophilic so traditionally the solvent Cremophor-EL is used to encapsulate it, however this is associated with toxic side effects and decreases the efficacy of PTX. Nanoparticles offer new methods of delivering this drug.

Abraxane (also known as Nab-PTX) uses a combination of NPs with albumin-bound PTX and has been approved by the food and drug administration (FDA) and European medicines agency (EMA) for the treatment of advanced breast cancer, pancreatic cancer, and NSCLC (15). For the treatment of NSCLC, Abraxane is administered intravenously at 100mg/m^2^ on days 1, 8 and 15 of a 21-day cycle; carboplatin is also administered on day 1 of the cycle (16) Abraxane has been shown to have a higher tumor uptake compared to solvent based PTX. It is believed Abraxane reaches tumor sites through the EPR effect and receptor-mediated transcytosis. When administered, the albumin-bound PTX binds to albumin-specific receptors such as glycoprotein 60 which activates caveolin-1 resulting in the formation of transcytosis vesicles. The vesicles are transported through the vascular endothelial cells and to the tumor tissue (17). Abraxane allows higher doses of PTX to be administered to patients with fewer side effects and decreased administration time (18).

Genexol-PM also contains PTX but uses a formulation of polymeric micelles. It has been approved in South Korea for the treatment of recurrent and metastatic breast cancer and NSCLC. It has been reported to have a maximum tolerated dose of 180mg/m^2^ when administered weekly to patients with solid tumors (19). A phase II study of patients with NSCLC showed that treatment with both genexol-PM and cisplatin had a significantly greater antitumor effect and allowed higher doses of PTX to be administered without significantly increasing toxicity (20). Treatment of NSCLC with genexol-PM and gemcitabine in a phase II trial also showed enhanced antitumor activity, however, severe side effects were frequently observed so further studies are needed to evaluate the safety of this treatment (21).

**Figure 2:**
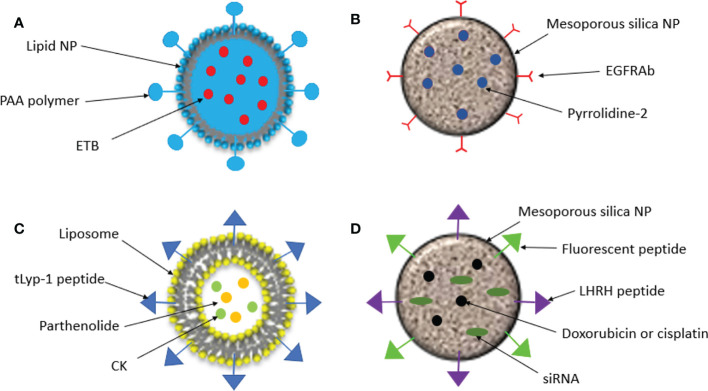
Schematic representation of nanoparticles created for targeted drug delivery in NSCLC. **(A)**. Lipid NPs were conjugated with the pH sensitive polymer, poly (acrylic acid) (PAA) and loaded with the kinase inhibitor Erlotinib (ETB). **(B)**. EGFR antibodies (EGFRAbs) were conjufated to silica NPs which were loaded with pyrrolidine-2, an inhibitor of cytosolic phospholipase A_2_ α, cPLA2α. **(C)**. Liposomal NPs were coated in tLYP-1 peptide and loaded with parthenolide and compound K (CK). **(D)**. Silica NPs were loaded with doxorubicin or cisplatin and an siRNA and an LHRH peptide was conjugated to target the tumor and a fluorescent protein was conjugated to measure fluorescence intensity.

Currently, 4 clinical trials utilizing nanoparticles for drug delivery in the treatment of NSCLC have reached phase III/IV, with one having completed – lipoplatin (22, 23). These are summarized in **Table 1** and **Figure 2**. Lipoplatin is a liposomal formulation of the chemotherapy agent cisplatin, which has been studied in the treatment of several cancers and has successfully completed phase I, II and III clinical trials for the treatment of NSCLC. Lipoplatin cannot be detected by immune cells so is able to circulate in the body for longer and due to the EPR effect it will preferentially accumulate in tumor tissue (22). A meta-analysis of clinical trials looking at the efficacy and safety of lipoplatin compared to conventional cisplatin, showed that patients with NSCLC had higher response rates to lipoplatin and this formulation had significantly lower toxicity than cisplatin. However, overall survival and progression free survival were not reported in most of these clinical trials so further studies are needed to confirm the benefit of lipoplatin compared to cisplatin (23).

**Table 1:** Nanoformulations approved or in phase III/IV clinical trials for the treatment of NSCLC. Adapted from Holder et al. (24).

Product/Clinical trial identifier	Active drug loaded	NP type	Clinical status	Reference
Abraxane	PTX	Albumin-bound combination	Approved for treatment of breast cancer, NSCLC, and pancreatic cancer	(15)
Genexol-PM	PTX	Polymeric micelles	Approved for treatment of breast cancer and NSCLC in South Korea	(19–21)
Lipoplatin	Cisplatin	Liposome	Completed phase III clinical trials for treatment of NSCLC	(22, 23)
NCT04033354	HLX10 Nab-PTX	Albumin-bound combination	Ongoing phase III clinical trial for treatment of NSCLC	(25)
NCT02667743	PTX	Polymeric micelles	Ongoing phase III trial for treatment of NSCLC	(26)
Lipusu/NCT02996214	PTX	Liposome	Ongoing phase IV trial for treatment of NSCLC	(27)

An active phase III trial is assessing the safety and efficacy of treatment with carboplatin and a Nab-PTX formulation similar to Abraxane together with the monoclonal antibody, HLX10, for stage III and IV NSCLC compared to carboplatin Nab-PTX treatment alone. HLX10 targets programmed cell death protein 1 (PD-1), which is involved in inhibiting the immune response and elevated levels have been observed in NSCLC (28). The expected completion date of this study was January 2023 (25)

A second ongoing phase III clinical trial uses a NP micellar formulation of PTX similar to Genexol-PM, in combination with cisplatin for the treatment of stage III/IV NSCLC (26) This study has demonstrated a significant improvement in overall rate response (50% compared to 26%) and progression free survival (an increase of 1.1 months) in patients treated with the polymeric micellar formulation of PTX and cisplatin compared to those treated with solvent based PTX and cisplatin (29). Although this trial has concluded, outcome measures are still being monitored.

Finally, a phase IV study is comparing the efficacy and safety of treatment of advanced NSCLC with a liposomal formulation of PTX (Lipusu) in combination with cisplatin and the standard treatment of cisplatin and gemcitabine (27). Treatment with Lipusu and cisplatin showed similar efficacy to the standard treatment but had a better safety profile with fewer treatment terminations and significantly lower incidences of adverse effects (30) EPR is a unique property of NPs that makes them suitable as drug delivery systems, however, relying on the effects of EPR alone only results in a moderate increase in drug delivery to tumors compared to healthy cells; therefore other methods are required to more effectively target tumor cells (31).

## Using NPs for targeted drug delivery

Another property of tumor tissues which can be exploited in targeted drug delivery is the slightly acidic environment that is observed in the tumor microenvironment (pH of 6.5 to 6.8). Nanoparticles can therefore be engineered to be pH-sensitive so that they only release anti-cancer drugs when they reach the tumor site (32) Tan and Wang, 2017 conjugated the pH-sensitive polymer, poly (acrylic acid) (PAA), to lipid nanoparticles which were loaded with the kinase inhibitor Erlotinib (ETB). *In vitro* cytotoxicity was significantly increased in A549 cells treated with the PAA-ETB-NP conjugate compared to ETB-NPs and ETB alone – suggesting the addition of PAA caused enhanced drug delivery to the tumor. Tumor inhibition was also measured in lung cancer mouse models. After 21 days tumor inhibition rate of PAA-ETB-NPs was significantly higher than ETB-NPs and ETB solution (84.5% compared to 68.7% and 38.1% respectively). Both ETB-NP conjugates had a much higher drug concentration in tumor tissue compared to the ETB solution and showed a far lower concentration in healthy tissues such as kidneys and the heart (33). This study demonstrates how drugs can be targeted to tumors using pH-sensitive release methods to increase drug delivery to tumors and decrease the effects of the drug in healthy tissue.

As discussed, patients with NSCLC are commonly tested for mutations in EGFR, ALK and KRAS, to predict which treatments patients will respond best to. Due to the overexpression of EGFR in many cases of NSCLC, it can be used for specific recognition of cancer cells. EGFR antibodies (EGFRAb) can be conjugated with NPs to direct them to tumor sites for targeted drug delivery. Sundarraj et al., 2014 conjugated EGFRAb to a type of hollow mesoporous silica nanoparticle called silica nanorattles (SN) and loaded this with pyrrolidine-2 (34). Pyrrolidine-2 is an inhibitor of cytosolic phospholipase A_2_ α (cPLA2α), an enzyme that catalyzes the hydrolysis of phospholipids to arachidonic acid and lysophospholipids, precursors of numerous biologically active lipids. In lung cancer, cPLA2α is believed to promote tumor growth by enhancing cell viability and proliferation (35). The internalization of the EGFRAb conjugated SN loaded with pyrrolidine-2 (EGFRAb-SN-pyrrolidine-2), was almost double in NSCLC cells compared to healthy lung cells (44.57 and 29.28% respectively) and *in vitro* studies showed that cytotoxicity, cell cycle arrest and apoptosis were significantly higher in cells treated with EGFRAb-SN-pyrroldine-2 compared with free pyrroldine-2 and SN-pyrroldine-2. *In vivo* studies with healthy mice found that EGFRAb-SN-pyrroldine-2 had low systemic toxicity compared with free pyrroldine-2, and in lung cancer mouse models EGFRAb-SN-pyrroldine-2 had an enhanced tumor inhibition rate. These findings demonstrate the ability of EGFRAb-SN-pyrrolidine-2 to effectively target drug delivery to lung cancer cells and enhance the effects of the drug and reduce toxicity to healthy cells.

Another protein which is often overexpressed in lung cancer is vascular endothelial-derived growth factor (VEGF). VEGF is the key inducer of angiogenesis through binding to VEGF receptor 2 (VEGFR2) and activating downstream signaling pathways. In many cancers VEGF is upregulated by oncogenes and tumor hypoxia leading to an overexpression of VEGFR2, promoting angiogenesis and therefore tumor growth (36). Neuropilin1 (NRP1) is a glycoprotein receptor which can act as a co-receptor with VEGFR2 and VEFA-165 to enhance their binding and promote angiogenic signaling (37, 38). NRP1 has been shown to be highly expressed in NSCLC is believed to correspond to poor patient prognosis, it is therefore a potential target for tumor therapy (39).

Jin et al., 2018 designed a liposomal NP which targeted NRP1 by coating the NP with the tumor-homing peptide, tLyp-1. They then loaded these NPs with parthenolide and ginsenoside compound K (CK), naturally occurring compounds which have shown numerous antitumor effects in cancers including NSCLC (40, 41). Cellular uptake of tLyp-1-liposomes was greater than liposomes coated in polyethylene glycol (PEG), and there was a greater accumulation of tLyp-1 in tumors from NSCLC mouse models. NSCLC cells exposed to CK/parthenolide tLyp-1 liposomes had significantly higher rates of apoptosis than those exposed to the free drugs alone or in combination. *In vivo* antitumor efficacy was also greater in mice treated with the CK/parthenolide tLyp-1-liposome formulation compared to the free drugs and PEG-liposomes loaded with the drugs. Lastly, lower systemic toxicity was observed in tissues of mice treated with the CK/parthenolide tLyp-1-liposomes compared to other treatments (42). This study suggested the CK/parthenolide tLyp-liposomes could effectively inhibit tumor growth and minimize the harmful effects of the drugs on healthy tissue.

Conventionally, anti-cancer drugs are administered intravenously meaning the drug will circulate around the body and affect healthy tissue. For treating NSCLC, delivery of anti-cancer agents *via* inhalation holds a number of advantages. Firstly, higher concentrations of the drug can be achieved at the tumor site so lower doses can be used, reducing systemic exposure and adverse effects. The alveoli of the lungs have a large surface area, so drugs are absorbed quicker and there is enhanced drug bioavailability as unlike the gastrointestinal tract, the lungs have a reduced enzyme activity relating to drug metabolism (43, 44).

Taratula et al., 2010 developed a mesoporous-silica nanoparticle (MSN) for drug delivery by inhalation for the treatment of lung cancer (45). The MSN was loaded with an anticancer drug (doxorubicin or cisplatin) as well as small inhibitory RNAs (siRNAs) targeted to either MRP1 or BCL2 mRNA in order to suppress drug resistance. The MSN-drug-siRNA conjugate was targeted to lung cancer cells by conjugating a luteinizing hormone-releasing hormone (LHRH) peptide, whose receptor is overexpressed in many cancer types including lung cancer (46) The MSN-drug-siRNA conjugate was fluorescently labelled, and fluorescence intensity was measured in different organs three hours after the drug was administered to lung cancer mouse models either by inhalation or intravenously. The amount of the MSN complex retained in the lungs was 14.6 times higher when it was administered by inhalation compared to intravenously. Accumulation of the complex in other organs was significantly decreased when administered by inhalation, limiting exposure to healthy tissue. *In vitro* cytotoxicity was measured, and the effect of the drugs was highest when delivered by the MSN complex in combination with siRNAs. This study demonstrates that nanoparticles can be used to effectively deliver drugs to the lungs through inhalation and limit exposure of the drug to healthy tissue.

Although NPs have been shown to have great potential in cancer treatment, there are still many issues that must be overcome before they can be widely used. The EPR effect which NPs rely on to target tumor sites, is random in nature and cannot be controlled. It is estimated that less than 1% of NPs that are injected reach the target site and the amount reaching healthy cells is substantial – causing therefore it is essential for tumor targeting to be optimized (47, 48). Another challenge with using NPs in the clinic is the effect of the immune response when NPs are administered. When NPs are administered, they can trigger the immune system and subsequently be destroyed through the immune response. Pre-clinical cancer studies often use immunocompromised mice, which may explain why success in these trials is not always translated when they are administered to humans in clinical trials (49) It is possible to engineer NPs to avoid recognition by the immune system. The size, shape and surface property of a NP will determine how the immune system responds; smaller, tubular, and inorganic NPs have been found to have better delivery efficiency (50). One way for NPs to evade the immune response is by coating them in a natural cell membrane. This has been demonstrated using red blood cell membrane coated NPs. The coated NPs retain many of the markers on the surface of natural red blood cells such as CD47, a protein that interacts with a receptor on macrophages, triggering a pathway that results in reduced phagocytosis (51) These coated NPs were able to evade the immune system and had an increased blood circulation time in mouse models (52).

## Discussion

Lung cancer has one of the poorest survival outcomes of all cancers with traditional therapies often resulting in high rates of reoccurrence and damage to healthy tissue causing numerous adverse effects. NPs offer enhanced methods of drug delivery to target sites and can improve the pharmacokinetics and pharmacodynamic profiles of pre-existing cancer drugs. Using NPs, drugs can be transported to tumor sites at higher concentrations without increased systemic toxicity. Approved nanoformulations and those in late stages of clinical trials, tend to rely on the EPR effect to achieve enhanced drug delivery to tumor sites; however, the benefits of this type of therapy are limited. To enhance the effects, the surface of nanoparticles can be modified so that they are targeted to cancer cells. This can be done by taking advantage of the slightly acidic environment of tumors and the overexpression of specific receptors that are seen in a number of cancers. By targeting cancer cells, it is possible to further improve drug efficacy and minimize the exposure of the drug to healthy tissues and decrease toxic side effects. NPs can also be adapted to allow them to be administered by inhalation, this offers a promising non-invasive alternative to intravenous cancer therapy and can enable lower doses of the drug to be administered whilst still achieving the same concentration in the lungs. Despite the promising advancements in nanotechnology, only a handful of nanoformulations created for cancer treatment have shown efficacy in clinical trials. This highlights the need for future research to enhance the targeting capabilities of NPs and improve uptake in tumors.

## Author contributions

JH, EO, CL, CT, LB, EB and CW contributed to conception and design of the study. JH and CW wrote the first draft of the manuscript. All authors contributed to the article and approved the submitted version.
